# Effects of Remote Ischemic Conditioning on Hand Engagement in individuals with Spinal cord Injury (RICHES): protocol for a pilot crossover study

**DOI:** 10.12688/f1000research.52670.2

**Published:** 2022-03-07

**Authors:** Yu-Kuang Wu, Noam Y. Harel, Jill M. Wecht, Ona E. Bloom

**Affiliations:** 1 Rehabilitation and Human Performance, Icahn School of Medicine at Mount Sinai, New York, NY, 10003, USA; 2 Bronx Veterans Medical Research Foundation, Bronx, NY, 10468, USA; 3 Department of Neurology, Icahn School of Medicine at Mount Sinai, New York, NY, 10003, USA; 4 The Feinstein Institute for Medical Research, Manhasset, NY, 11030, USA; 5 The Zucker School of Medicine at Hofstra Northwell, Hempstead, NY, 11549, USA

**Keywords:** Spinal cord injury, Remote ischemic conditioning, corticospinal, TLR pathway, neural plasticity

## Abstract

​​​​​​ **Background**: Most spinal cord injuries (SCI) are not full transections, indicating that residual nerve circuits are retained. Rehabilitation interventions have been shown to beneficially reorganize motor pathways in the brain, corticospinal tract, and at the spinal level. However, rehabilitation training require a large number of repetitions, and intervention effects may be absent or show transient retention. Therefore, the need remains for an effective approach to synergistically improve the amount and duration of neuroplasticity in combination with other interventions. Remote ischemic conditioning (RIC) demonstrates several potential advantages as a candidate for such an approach. Therefore, we propose a protocol to investigate RIC coupled with physical training to promote neuroplasticity in hand muscles.

**Methods**: This will be a prospective randomized-order crossover trial to be performed in eight able-bodied participants and eight participants with chronic cervical SCI. Patients will participate in two experimental sessions consisting of either active or sham RIC preceding a bout of pinch movement exercise. Serial evaluations will be conducted at baseline, after RIC, immediately after pinch exercise, and follow up 15-minutes later. The primary outcome is the change in corticospinal excitability (primarily measured by the motor evoked potential of abductor pollicis brevis muscle). Secondary outcomes will include maximal volitional pinch force, and inflammatory biomarkers. To ensure safety, we will monitor tolerability and hemodynamic responses during RIC.

**Discussion**: This protocol will be the first to test RIC in people with cervical SCI and to investigate whether RIC alters corticospinal excitability. By sharing the details of our protocol, we hope other interested researchers will seek to investigate similar approaches – depending on overlap with the current study and mutual sharing of participant-level data, this could increase the sample size, power, and generalizability of the analysis and results.

**Trial registration**: ClinicalTrial.gov, ID: NCT03851302; Date of registration: February 22, 2019

## Introduction

### Background and rationale

Between 250-350,000 individuals live with chronic spinal cord injury (SCI) in the United States. Among this population, ~60% have injuries at the cervical level ^1^. Impairments of arm and hand function in individuals with cervical SCI greatly reduce quality of life and adversely impact the level of independence ^2^. Previous research on the needs of individuals with cervical SCI has shown that improvement of hand function is ranked more important than walking ^3^. Most spinal cord injuries are not full transections, indicating that functional nerve circuits exist after injury ^4^. Rehabilitation interventions such as physical training and neural stimulation have been shown to reorganize motor pathways in the brain, corticospinal tract, and at the spinal level to promote functional gains ^5– 8^. Neural activation drives neuroplasticity. Thus, enhancing residual neural circuit excitability after SCI could improve hand function in the long term by increasing neuroplasticity ^9– 14^.

However, even a large number of repetitions of physical training often leads to transient, poorly retained benefits, and some individuals with SCI remain non-responders. Investigators must find new ways to enhance the magnitude and duration of neuroplastic effects. Acute intermittent hypoxia (AIH) is one such approach. Preliminary research has shown that AIH coupled with task-oriented physical rehabilitation enhances the learning effects of task-specific motor training after SCI ^15, 16^, presumably through activation of raphe serotonergic neurons ^17^. This leads to intermittent spinal serotonin release, which further induces synthesis of brain-derived neurotrophic factor (BDNF) and activation of the high-affinity receptor tyrosine kinase, potentially leading to neuroplasticity ^18^. However, AIH requires a hypoxicator system to provide systemic low oxygen exposure, and highly trained staff are needed to administer AIH, making it difficult for home use. Therefore, a need remains for developing an accessible, low-risk method to promote lasting neuroplasticity and potentially be used in the home setting.

Ischemic conditioning occurs when a specific organ or tissue is exposed to one or more transient episodes of sublethal ischemia. This leads to several reactions that protect the organ system or tissue from subsequent ischemia ^19– 21^. Murry *et al.* first introduced this technique in 1986 ^22^, showing that a brief cycle of occlusions and reperfusion to the coronary artery reduced the size of myocardial infarction in the canine heart. Subsequent studies have demonstrated that these endogenous protective effects are not limited to the heart or the ischemic organ/tissue alone – the protective effects are systemic and they are transferrable to other organ systems or tissues ^23– 25^. This phenomenon is called remote ischemic conditioning (RIC) ^26, 27^, and for example, cardioprotective effects have been reported by simply occluding arterial blood flow to one upper extremity for 3-5 cycles of 5 minutes each using a tourniquet. Additionally, the neuroprotective effect of RIC has been shown in animal studies. Studies in rats and rabbits have shown that RIC can protect the spinal cord from acute ischemia-reperfusion injury ^28– 31^, possibly through upregulation of antioxidant enzyme activity, alteration of endocannabinoids system, or modulation of heat shock protein expression. Among the potential mechanisms supporting the cardioprotective benefits of RIC, two might promote neuroplasticity: induction of trophic and anti-inflammatory factors. Hypoxia-inducible factor 1α (HIF-1α) may have neuroprotective effects via triggering the expression of genes related to oxygen transport, glycolytic metabolism, and apoptosis ^32^. Albrecht and colleagues found that upper limb RIC induced H1F-1α accumulation and activation in right atrial tissue of patients undergoing cardiopulmonary bypass ^33^. Vascular endothelial growth factor (VEGF) is another potentially neuroprotective factor shown to be induced by RIC ^34^. Whether the activation of H1F-1α and VEGF induced by RIC extends to corticospinal areas and regulates neural excitability is unknown.

On the other hand, RIC may reduce systemic inflammation ^35, 36^. The systemic inflammation has been shown to attenuate expression of BDNF in the brain ^37– 40^. One study demonstrated broadly elevated inflammatory gene expression in persons with chronic SCI, which was particularly evident in persons with higher level injuries ^41^. Specifically, elevated expression of members of the Toll-like receptor 2/4 (TLR) signaling pathway, metalloproteinases (ADAM10), caspases (CASP1, 3, 8) and chemokine gene families were identified in individuals with SCI compared to able-bodied individuals. One study in four able-bodied adults showed that RIC (by inflating pressure cuff on forearm to 200 mm Hg for three cycles of 5-min occlusion and 5 min reperfusion) reduced proinflammatory gene expression in circulating leukocytes 15 minutes and 24 hours later ^35^. Of interest, members of the same genes and gene families elevated in persons with chronic SCI ^41^ were reduced by RIC in able bodied individuals: TLR signaling pathway, Tumor Necrosis Factor (TNF) receptor pathway, Mitogen-Activated Protein (MAP) kinases, apoptosis pathway (CASP8), chemokines, T cell signaling molecules, metalloproteinases and leukocyte adhesion moleculesref ^35, 41^

Cherry-Allen and colleagues recently published the first study testing the synergistic effects of RIC on motor task learning ^42^. Able-bodied adults (n=18) were randomly assigned into active or sham RIC groups to undergo seven consecutive weekday sessions. Active or sham RIC (five cycles of 5-min inflation and 5-min deflation by upper arm blood cuff inflation to 200 mm Hg in active RIC and 10 mmHg below the subjects’ diastolic blood pressure in sham RIC) was conducted before training each day. Daily training consisted of stability platform balance training and training of a hippocampal-dependent cognitive task. The authors noted significantly improved performance on the stability platform task in the active RIC group compared to the sham group immediately after the completion of seven-day training programs; these effects were retained at the 2- and 4-week follow-up visits. The authors hypothesized that the improved performance might be due to increased BDNF production by hippocampal and corticospinal neurons induced by RIC. However, they did not report significant changes in serum BDNF, cognitive learning, or generalized muscle activation measured by finger flexor EMG activity and grip force in the RIC compared to the sham group. Corticospinal excitability was not directly measured in that study. Therefore, the mechanism of improved balance was unclear. We hypothesize that RIC acutely modulates corticospinal excitability as measured by utilizing transcranial magnetic stimulation (TMS).

Although RIC has been shown to be safe in the healthy able-bodied population as well as in individuals with heart disease and critically ill patients with subarachnoid hemorrhage ^43– 46^, there are no data describing the safety of RIC in the SCI population. However, widespread sensory impairment, including a limited ability to feel pain/discomfort, may compromise the safety profile of such techniques in participants with SCI. In addition, damage to the autonomic nervous system (ANS) after cervical SCI contributes to cardiovascular dysregulation, which may alter hemodynamic responses to RIC. Several studies ^47– 50^ have reported stable heart rate (HR) and blood pressure (BP) responses before and after RIC in healthy participants, and also in participants with heart disease or vascular stenosis. However, sympathetic hypoactivity after cervical SCI might result in altered or delayed hemodynamic responses, possibly leading to fluctuation of HR and BP during RIC. These possibilities make it essential to investigate HR, BP and oxygen saturation (SaO _2_) in real-time and document acute pain/discomfort and any other adverse effects during RIC in individuals with cervical SCI.

Therefore, we propose a proof of concept study to investigate the acute synergistic effects of active versus sham RIC with motor task training (isometric hand exercise) in persons with chronic SCI. The primary outcome measure will be change in corticospinal excitability. Secondary outcomes will include pinch force and inflammatory biomarkers specifically on TLR signaling pathway. To ensure safety, we will monitor beat-to-beat changes in HR, BP and SaO _2_ during RIC, since ANS damage in individuals with SCI might dysregulate hemodynamic responses during ischemic conditioning. This protocol details our experimental design and explores the possible benefits of RIC on neuroplasticity. An important advantage of RIC is that it can be simply applied using a regular blood pressure monitor and a timer. Therefore, if a beneficial effect of RIC on neuroplasticity is demonstrated, RIC can be easily coupled with rehabilitation training in the clinic or home setting. 

As far as we know, this will be the first study in the SCI population to (1) investigate the synergistic effects of RIC with physical training on corticospinal excitability, (2) measure changes in inflammatory mediators after RIC, and (3) observe in real time the responses of HR, BP and SaO2 during RIC.

### Objectives

*Aim 1: To determine the effects of active versus sham RIC prior to one bout of muscle contraction exercise on motor corticospinal excitability*.

- *Hypothesis 1A:* Motor evoked potential (MEP) amplitudes at the abductor pollicis brevis (APB) muscle (Primary Outcome) will significantly increase after active RIC plus isometric hand exercise compared to sham RIC plus isometric hand exercise. Active RIC alone will not significantly increase motor evoked potential amplitudes. These trends will be similar in both SCI and able-bodied participants.
- *Hypothesis 1B:* Secondary electrophysiological outcomes at the APB muscle will significantly change after active RIC plus isometric hand exercise compared to sham RIC plus isometric hand exercise. Short-interval and long-interval cortical inhibition will decrease; intracortical facilitation will increase. Active RIC alone will not significantly change these outcome measures. These trends will be similar in both SCI and able-bodied participants.

*Aim 2: To investigate effects of active versus sham RIC on systemic inflammatory mediators in individuals with cervical SCI*.

- *Hypothesis 2A:* The gene expression of inflammatory mediators will significantly decrease after active RIC compared to sham RIC in participants with SCI.
- *Hypothesis 2B:* The trends of inflammatory mediator change will be similar in both participants with SCI and able-bodied participants.

*Aim 3: To determine changes in heart rate (HR), blood pressure (BP) and oxygen saturation (SaO _2_) during active versus sham RIC in individuals with cervical SCI and able-bodied subjects*.

- *Hypothesis 3A:* There will be no difference in HR, BP and SaO _2_ (pulse oximeter on the finger of the target arm) responses among baseline, inflation phase, deflation phase and post-RIC during active and sham RIC in persons with SCI and able-bodied controls.

### Trial design

**Figure 1.**
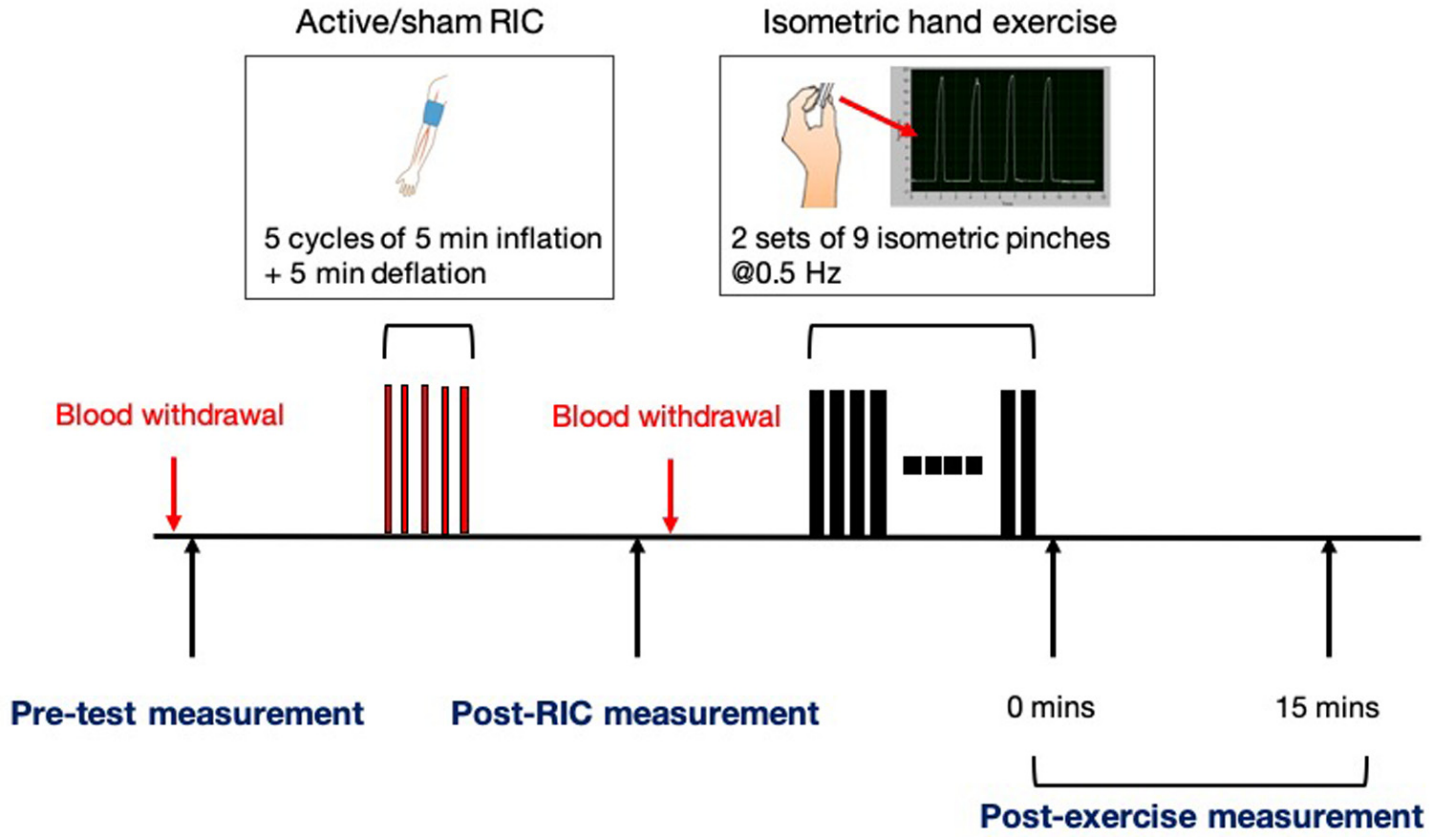
The experimental protocol.

This is an exploratory prospective randomized-order crossover trial to be performed over 24 months in 16 participants, including eight able-bodied participants and eight participants with cervical SCI. In two separate sessions, active or sham RIC will be performed prior to isometric hand exercise. The reason to enroll able-bodied participants is to investigate corticospinal excitability and ANS responses as a reference comparing to SCI, because this is the first study to test the synergistic effects of RIC with physical training on corticospinal excitability in either able-bodied or SCI subjects. Participants with SCI will undergo a screening session prior to the first experimental session to determine eligibility. Participants will be randomly assigned the order of the two experimental sessions: active or sham RIC. The isometric hand exercise will be performed in both sessions. Clinical and physiological measurements will be made during each session before and after sham/active RIC, and after isometric hand exercise ( Figure 1). Blood samples will be drawn before and after sham/active RIC. The washout period between the two experimental sessions will be at least 14 days to prevent any carry-over effects.

## Methods: participants, interventions and outcomes

### Study setting

Single-center (James J. Peters VA Medical Center (JJPVAMC); Bronx, NY, USA); a clinical research center within government-academic hospital.

### Eligibility criteria

***Inclusion criteria.*** Participants with SCI will be included if they fulfill the following inclusion criteria: (1) age between 18 and 65 years; (2) chronic (more than 12 months since injury) complete and incomplete SCI between neurological levels C2-C8 determined by the International Standards for the Neurological Classification of Spinal Cord Injury (ISNCSCI); (3) score of 3 or more (out of 5) on manual muscle testing of finger extension, finger flexion, or finger abduction in left or right hand; (4) detectable motor evoked potentials in left or right APB via TMS; (5) able to perform thumb-middle finger opposition pinch task with detectable pinch force. A screening session (details described in the section *Interventions*) will be conducted for each SCI participant to determine criteria (2) to (5).

Additionally, able-bodied participants between ages 18 and 65 years without any known central or peripheral neurological disease or injury will be recruited.

***Exclusion criteria.*** Participants will be excluded if they have any of the following: (1) multiple spinal cord lesions; (2) history of seizures; (3) use of medications that significantly lower seizure threshold, such as amphetamines and bupropion; (4) history of implanted brain/spine/nerve stimulators, aneurysm clips, or cardiac pacemaker/defibrillator; (5) any other contraindication to undergoing magnetic resonance imaging (except for claustrophobia); (6) significant coronary artery or cardiac conduction disease; (7) open skin lesions over the arms; (8) pregnant; (9) unsuitable for study participation as determined by study physician.

### Consent

***Who will take informed consent?*** The principal investigator and IRB-approved research coordinators will obtain informed consent. Explanations about the procedures are provided in a quiet private area in our research center at the JJPCAMC. This is done at the potential participant’s convenience. An explanation is given to them for releasing any identifiable information and they are assured that this information is kept in a securely locked cabinet. Only members of the investigative team have access to and may use the information.

***Additional consent provisions for collection and use of participant data and biological specimens.*** The informed consent and the form “Authorization for Use and Release of Individually Identifiable Health Information Collected for VHA Research” ( *Extended data* ^51^) specifically address the collection of blood samples for the analysis of inflammatory biomarkers and ask participants’ permission to save their data in a secure data repository for other research studies in the future. Samples will only be saved/analyzed for future studies if participants consent.

### Interventions

***Explanation for the choice of comparators.*** This study will test the short-term effects of active RIC, which is designed to occlude arterial blood flow to one arm for five cycles of 5 minutes each. The comparator (sham RIC) will use the same equipment (an inflatable blood pressure cuff), but the inflation pressure will be lower than active RIC (10mm Hg below diastolic blood pressure). As with active RIC, sham RIC will be delivered to one arm for five cycles of 5 minutes each. The sensations of sham RIC will be qualitatively similar to active RIC, but it is possible that participants will feel quantitatively less pressure in their arm. We will not inform participants ahead of time which sensation (higher versus lower pressure) is active versus sham. 

***Intervention description***

Screening session

The purpose of the screening session is to ensure that participants with SCI meet all inclusion/exclusion criteria including detectable electrophysiological signals induced by TMS. Subjects will first undergo neurological examination according to the International Standards for the Neurological Classification of Spinal Cord Injury (ISNCSCI). The ISNCSCI neurological level of injury must be between C2–C8, with muscle strength of at least 3/5 in finger extension, finger flexion, or finger abduction in either hand. In order to qualify for the study, all participants must demonstrate the capability of performing a pinch grip between the tips of the thumb and third finger with detectable pinch force on at least one hand. Maximal volitional contraction (MVC) during thumb-third finger pinch will be recorded using a load cell dynamometer. Supramaximal electrical stimulation will be delivered at median and ulnar nerves at wrist level to induce maximal peak-to-peak amplitude of M wave (Mmax) and F wave. Recorded peripheral nerve parameters include the latency and the peak-to-peak amplitude of the M/F waves. After determining each participant’s optimal scalp location for hand motor cortex stimulation using TMS (Magventure X100 with D-B80 coil) with neuronavigation (Brainsight), TMS will be performed to determine resting motor threshold (RMT), which will be defined as the lowest intensity at which at least 5 out of 10 stimuli result in a response of at least 50 uV from the targeted APB. If RMT cannot be detected at the APB muscle at stimulus intensity below 90% of maximal stimulator output, then active motor threshold (AMT) will be determined while the participant performs a pinch maneuver at 20% maximal effort. Participants will be ineligible for study participation if neither RMT nor AMT can be detected at the APB muscle at stimulus intensity below 90% of maximal stimulator output. If MEPs are detected, then an intensity-response curve will be recorded at 10–20% intervals between 100% and 200% of threshold in pseudorandom order. Peak-to-peak amplitude of 10 responses will be averaged per intensity. To account for possible changes in electrode placement and skin conductance over different testing sessions, MEPs will be normalized during each session to peripherally evoked Mmax ^52^.

Study session

Figure 1 depicts the experimental protocol. Each session will consist of a pre-test measurement (baseline), active or sham RIC, post-RIC measurement, an isometric hand exercise and post-exercise measurements. Blood samples (3cc) will be collected before the active/sham RIC cycle and 15 minutes after completion of active/sham RIC to measure changes in inflammatory mediators.

The RIC protocol involves five cycles of 5-min inflation and 5-min deflation on the non-target arm. The cuff will be placed above the elbow crease in similar fashion as used to measure brachial artery blood pressure via a personal tourniquet system (Delfi PTS Personal Tourniquet System or Blood Flow Restriction). The original RIC protocol used 200 mmHg inflation ^26, 43, 44, 53– 55^; however, most individuals with cervical SCI are hypotensive, and may not need this high of a pressure for effective RIC. Cuff inflation to 20 mmHg above systolic blood pressure has been shown to have the same ischemic effects as 200 mmHg inflation pressure ^56^. Therefore, in order to increase tolerability:

- **active RIC** will be performed at a cuff inflation pressure of 20 mmHg above each participant’s resting systolic blood pressure and
- **sham RIC** will be performed at 10 mmHg below each participant’s diastolic blood pressure.

The RIC placement and procedure is directly overseen by the principal investigator (YW, a licensed physical therapist) and co-investigator (NYH, a licensed neurologist).

**Figure 2.**
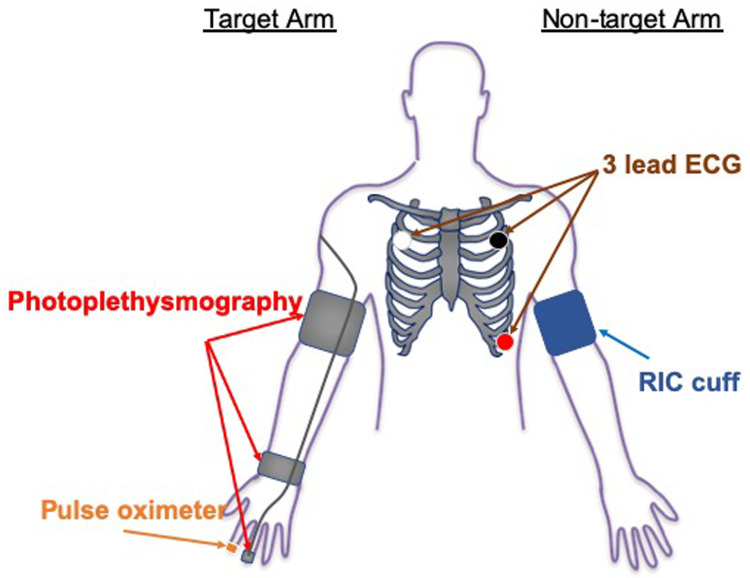
The equipment configuration for testing HR variability, respiratory rate and blood pressure changes during RIC/Sham conditioning.

During active and sham RIC, beat-to-beat HR, BP and SaO _2_ will be monitored in real-time on the contralateral limb and digital signals will be stored on a computer hard-drive for subsequent analysis. A 3-lead ECG (UFI: model RESP 1, Morro Bay CA) will be used to monitor beat-to-beat HR during testing. Electrodes will be placed at the right and left clavicle and in the V-5 position; data will be recorded from V-5. Beat-to-beat BP will be continuously monitored from the target middle or ring finger using photoplethysmography (FMS: Finometer Pro; Amsterdam, Netherlands). Continuous SaO _2_ will be monitored with a finger pulse oximeter, placed on the contralateral hand, which will be recorded at 1-minute intervals during administration of the active and sham RIC conditions ( Figure 2 shows the equipment configuration during RIC/Sham conditioning). At the fourth minute of each inflation period, participants will be asked to report any pain (on a scale from 0 to 10), discomfort, or other symptoms. Participants will be asked again at the first minute of the following deflation period to check if the pain, discomfort, or other symptoms persist.

Several studies have demonstrated transient increased MEPs as an immediate result of post-exercise facilitation in able-bodied participants after a short period of repetitive contraction exercise in thenar ^57^, wrist ^58^, forearm ^59^, or leg muscles ^60, 61^. Reports suggest that performing volitional movements at varying intensities further stimulates corticospinal circuits ^62, 63^. As such, for the isometric hand exercise, participants will be instructed to pinch a dynamometer with tips of the thumb and third finger at different intensities and durations. The various combinations of intensity and duration of pinch movements in our study, as well as the use of an intrinsic hand muscle, theoretically involves more cortical attention, which should magnify the likelihood of modulating supraspinal neuroplasticity ^62– 64^. Pinch force intensities will be randomized between 10%, 25%, and 50% of MVC, and the duration of contraction at each intensity will vary between durations of 2, 4, and 6 seconds, which results in nine different combinations delivered in pseudo random order. Participants will perform 2 sets of the 9 combinations of isometric hand exercise (18 pinches in total). The interval between each pinch task will be 2 seconds, with 30 second intervals between each set. A video in the following Figshare link demonstrates how the subjects perform the isometric hand exercise in the study protocol: https://figshare.com/articles/media/RIC_study_Pinch_exercise_video/16618132

Outcomes will be measured at baseline, post-active/sham RIC (post-RIC), and post-exercise (immediately after the isometric hand exercise (post-RICx-0) and 15 minutes later (post-RICx-15)) ( Figure 1).

***Criteria for discontinuing or modifying allocated interventions.*** If there are any major problems, such as significant change in blood pressure, or shortness of breath, or other significant discomfort during the screening visit and experimental visits, the procedure will be immediately halted, appropriate medical care will be provided, and for the participants’ safety, they will not be eligible for further participation in the study.

***Strategies to improve adherence to interventions.*** The study is three sessions for SCI participants, and two sessions for able-bodied volunteers. Prior to enrolling, we will explain the time commitment necessary. Potential enrollees who cannot firmly commit to the time necessary will not be enrolled.

### Relevant concomitant care permitted or prohibited during the trial

Participants will not be required to halt any drug or other concomitant care interventions during trial participation.

### Provisions for post-trial care

The study site, JJPCAMC, will provide necessary medical treatment in accordance with applicable federal regulations to a research subject injured by participation in a research project approved by a VA R&D Committee and conducted under the supervision of one or more VA employees.

### Outcomes

***Electrophysiological outcomes.*** The primary muscles assessed in this study are APB and the first dorsal interosseous (FDI) muscle on the target hand.

Primary outcome:

*Peak to peak amplitude of motor evoked potentials at 120% intensity of motor threshold (MEP _120_):* A winged TMS coil will be positioned over the optimal scalp location corresponding to the motor cortex area innervating the APB muscle of the target hand. The single-pulse TMS protocol to measure MEP _120_ amplitude comprises 10 unconditioned stimuli elicited at a stimulus intensity of 120% of the resting (or active) motor threshold as identified in the screening session. The MEP _120 _is a simple variable to measure the change of corticospinal excitability that has been used in multiple paired pulse and plasticity experiments ^65– 67^. The 10-second inter-pulse interval has been shown to have good reliability ^68^. To account for possible changes in electrode placement and skin conductance over different testing sessions, MEPs are normalized during each session to peripherally evoked Mmax as previously described ^52^.

Secondary outcomes:

*Recruitment curve:* Also called input-output or stimulus-response curve, this indicates the increase in MEP amplitude with increasing TMS intensity. The recruitment curve is assumed to be associated with the strength and excitability of corticospinal projections ^69^. In our protocol, the stimulus-response curve will be recorded at 10–20% intervals between 90% and 200% of threshold in pseudorandom order. Recruitment curves representing the relationship between stimulation intensity and induced MEP amplitude will be fit using a sigmoid function:

$$MEP\;=\;\frac{a}{1\;+\;\exp\;(-\frac{x\;–\;x0}{b})}$$

where **α** is the maximum MEP defined by the function; b is the slope parameter of the function; x is the stimulus intensity, and x0 is the stimulus intensity at which MEP amplitude is 50% of the maximum ^70^.

*Cortical inhibition and facilitation:* Paired-pulse TMS will be used to measure short interval cortical inhibition (SICI), long interval cortical inhibition (LICI) and intra-cortical facilitation (ICF) ^65^. Paired-pulse TMS includes a conditioning (CS) and test stimulus (TS) separated by a specified interstimulus intervals (ISI). The configuration for measuring SICI, LICI and ICF in this study are as follows ^71– 74^:

          SICI: CS = 90% RMT, TS = 120% RMT, ISI = 3 ms

          LICI: CS = 120% RMT, TS = 120% RMT, ISI = 100 ms

          ICF: CS = 90% RMT, TS = 120% RMT, ISI = 12 ms

Ten paired-pulses will be obtained for each condition with an 8 second interval between each paired-pulse.

*Peripheral nerve profile:* Supramaximal electrical stimulation will be delivered to the median and ulnar nerves at wrist level. The peripheral nerve profile includes the latency and the peak-to-peak amplitude of the M/F waves. The peak-to-peak amplitude of the M waves (Mmax) will be used to normalize the MEP _120_ at each time point to ensure that changes of corticospinal excitability are not due to variation in recording electrode placement ^65^.

***Functional outcomes.*** *Pinch force:* Maximal voluntary thumb-3 ^rd^ finger pinch force will be measured by a load cell dynamometer (100lb S-Beam load cell, ANYLOAD, New Jersey, USA) with customized 3D printed holder designed for pinch force measurement. Participants will perform three attempts of maximal pinch force and the best one will be recorded.

***Gene expression of inflammatory biomarkers.*** Housekeeping genes included in the array are ACTB, B2M, GAPDH, HPRT1 and RPLP0; the one most empirically stable will be used as the reference control gene for normalization. We will compare the changes of gene expression between baseline and after RIC.

***Hemodynamic stability during RIC.*** Beat-to-beat HR, BP and SaO _2_ will be collected in baseline, inflation phase, deflation phase and post-RIC.

The participant timeline can be seen in Table 1. The original table can be also found on the figshare website at the below link:

**Table 1.** Participant timeline.

	STUDY PERIOD			
	Enrolment	Allocation	Post-allocation	
TIMEPOINT	*0*		*t _1_*	*t _2_*
**ENROLMENT:**				
**Eligibility screen**	X			
**Informed consent**	X			
**Allocation**		X		
**INTERVENTIONS:**				
***Active RIC***			X	
***Sham RIC***				X
**ASSESSMENTS:**				
**Electrophysiological Outcomes**	X		X	X
**Gene expression of inflammatory biomarkers**			X	X
**Hemodynamic stability during RIC**			X	X

### Sample size

This project is the first to test RIC on people with SCI and investigate whether RIC alters nerve excitability in the pathway from the brain to the spinal cord to facilitate motor learning. For the primary efficacy outcome measure (MEP) of corticospinal excitability, we utilized G*Power 3.1 ^75^ to estimate the sample size of four groups (Condition: SCI-RIC, SCI-sham, AB-RIC, AB-sham) by four measurements (Time: baseline, post-RIC, post-RICx-0, post-RICx-15) within-between (mixed) ANOVA. A sample size of 16 with 80% power would be needed to detect a large effect size (Cohen’s f) of 0.4 at a significance level of 0.05. This pilot study emphasizing safety and feasibility will enroll 16 participants (8 SCI and 8 able-bodied) to more specifically determine the effect size for subsequent efficacy trials.

### Recruitment

Veterans will be recruited from the SCI Service and from ongoing research studies taking place at the JJPCAMC. After our veteran population has been given first option to participate, eligible non-veterans from the tri-state New York/New Jersey/Connecticut metropolitan area will be permitted to participate. This will allow for a larger pool of subjects and will help to ensure balanced allocation among treatment groups. Our research Center has enrolled almost 1,000 participants with chronic SCI in various clinical research studies over the past two decades.

Eligible women, minorities, and economically or socially disadvantaged individuals will be included. These individuals, potentially classified as vulnerable populations, will be given the same treatment as individuals who are not considered to be vulnerable. No special classes of subjects such as pregnant women, prisoners, or institutionalized individuals will be recruited for this study.

There will be no exclusions based on gender, race, or ethnic status. However, regarding gender, please note that approximately 80% of traumatic spinal injuries occur in males. Furthermore, although our research is open to non-veterans, the predominant component of our population at the VA is male.

### Assignment of interventions: allocation

***Sequence generation.*** A list of computer-generated random assignments using the numbers 0 (first session sham) and 1 (first session active) will be kept by a researcher uninvolved in this clinical trial. Random assignments will be distributed to each enrollee in sequence to determine the order of active and sham RIC that a participant will be receive.

***Concealment mechanism.*** The researcher holding the allocation sequence will be otherwise uninvolved in the trial. The next allocation will only be revealed upon enrollment of a new participant. Allocation concealment will be ensured, and the researcher will not release the randomization code until the participant has been recruited into the trial, which takes place after the baseline measurements have been completed. Therefore, neither the study coordinators, investigators, nor enrollees will know in advance which intervention sequence they will receive.

***Implementation.*** All participants who give consent for participation and who fulfill the inclusion criteria will be randomized. A list of computer-generated random assignments using the numbers 0 (first session sham) and 1 (first session active) will be kept by a researcher uninvolved in this clinical trial. Research coordinators for recruitment and study condition are not allowed to receive information about the group allocation.

### Assignment of interventions: blinding

***Who will be blinded.*** Assessments regarding outcome measurements will be conducted by the research coordinators blind to the intervention allocation. The research coordinators will go through a profound assessment training program. Due to the nature of the intervention, neither participants nor the research coordinators can be 100% blinded to allocation but are strongly inculcated not to disclose the allocation status of the participant during the experiment. A researcher outside the research team will randomize the order of active and sham RIC for each participant and configure the personal tourniquet system. The inflation pressure value will be concealed throughout the whole period of RIC. The researcher will feed data into the computer in separate datasheets so that the research team can analyze data without having access to information about the allocation.

***Procedure for unblinding if needed.*** Not applicable.

## Data collection and management

### Plans for assessment and collection of outcomes

***Electrophysiological outcomes.*** The primary muscles assessed in this study are APB and the FDI muscle on the target arm.

MEP _120 _and intra-cortical facilitation/inhibition (SICI, LICI and ICF) at the target APB and FDI muscles post-RIC, and at 0- and 15-minutes post-isometric pinch exercise will be expressed as percentage change compared to baseline. The primary outcome will be **MEP _120_** of APB muscle at the post-RICx-0 timepoint. **The MEP _120_** will first be normalized to peak-to-peak amplitude of M waves (Mmax).

***Gene expression of inflammatory biomarkers.*** RNA will be isolated from whole blood collected in PAXgene tubes (PreAnalytix, BD), using standard methods and the manufacturer’s protocol (Qiagen QIAcube, Venlo, The Netherlands). RNA quality and quantity will be determined using the Bioanalyzer (Agilent). RNA will be converted to complementary DNA (cDNA) (complementary DNA) using the RT ^2^ (reverse transcriptase) First Strand Kit and RT ^2^ SYBR Green Mastermix. We will use the PCR (Polymerase Chain Reaction) Array for Human Toll-Like Receptor Signaling Pathway (Qiagen, USA) on the Roche Lightcycler 480 (384-well block). Relative gene expression will be determined using the delta Ct method. Housekeeping genes included in the array are ACTB, B2M, GAPDH, HPRT1 and RPLP0; the one most empirically stable will be used as the reference control gene for normalization. The delta Ct method ^76^ will be used to determine relative gene expression. This standard method to quantify PCR products uses the cycle threshold (Ct) needed for the fluorescent signal derived from SBYR Green dye incorporation into the PCR product to increase above a background level (determined by a negative control, such as lack of cDNA included in the PCR reaction). The Ct is inversely proportional to the amount of target sequence in the substrate cDNA. The delta Ct is the Ct of the housekeeping or control gene minus the Ct of the gene of interest. The delta Ct method calculates the difference in the delta Ct values in two conditions, such as between a control (baseline) and an experimental time point.

***Hemodynamic stability during RIC.*** During the 50-minute active and sham RIC (five cycles of 5 min inflation plus 5 min deflation), beat-to-beat HR, BP and SaO _2_ will be collected. The peak HR, BP and the minimal SaO _2 _in baseline, inflation phase, deflation phase and post-RIC will be recorded for statistical analysis.

### Plans to promote participant retention and complete follow-up

The study is three sessions for SCI participants, two sessions for able-bodied volunteers. Prior to enrolling, we will explain the time commitment necessary. Potential enrollees who cannot firmly commit to the time necessary will not be enrolled. Outcome data collected for participants who discontinue from the protocol will still be analyzed.

### Data management

Every subject will have a recording sheet with de-identified data which will be kept in the source documents binder. Subject’s contact information and consent forms which contain personal health information (PHI) and a key linking subject numbers to their names will be stored separately from unidentified, linked data and this will be kept in locked file cabinet in a locked office in our Center, or on the firewall-protected VA network. Data will be subjected to error analysis through mechanisms such as range checks for invalid values.

### Confidentiality

Only study team members will have access to the Research materials obtained from participants during this Study. These materials will be secured in a locked file cabinet in the Center as well as on a password-protected file on the VA server. Participants’ identifiable and PHI will be protected by coding participants’ identity. Only the study team members will have access to the code. The code is kept in locked files and secure electronic servers. The code will not be used to link the information back to participants without their permission, unless the law requires it.

### Plans for collection, laboratory evaluation and storage of biological specimens for genetic or molecular analysis in this trial/future use

The blood samples will be de-identified and labelled with participants’ numbers (coded). The blood samples will be sent to the Feinstein Institute for Medical Research for inflammation biomarker analysis. The principal investigator is responsible for taking the biological specimens off-site, who is well qualified to handle and ship biohazardous materials at the JJPCAMC for handling, and has completed the training at the VA before handling the specimens. Blood samples will not be stored for future use.

## Statistical methods

All the statistical analyses will be conducted in IBM SPSS version 26.

### Statistical methods for primary and secondary outcomes

***Electrophysiological outcomes.*** Electrophysiological outcomes and pinch force will be compared after RIC and after the isometric hand exercise relative to baseline values. Descriptive analysis will be computed first for all outcome variables to test the distribution of the data and correlation tests will be performed to check the independence among outcome variables. A three way 2 (Group: cervical SCI, able-bodied) by 2 (Condition: RIC, sham) by 4 (Time: baseline, post-RIC, post-RICx-0, post-RICx-15) mixed-ANOVA will be used to analyze MEP _120_, intra-cortical facilitation/inhibition and pinch force. If the data is not normally distributed and the assumptions of the ANOVA are not met, Wilcoxon signed rank tests will be used instead. Post hoc pairwise comparisons will be performed using the Bonferroni adjustment to test significant pairwise comparisons following significant main or interaction effects among the three independent factors (Group; Condition; Time).

***Gene expression of inflammatory biomarkers.*** A three-way 2 by 2 by 2 mixed model ANOVA will be performed for 2 within group factors (Time: baseline vs. post- RIC) (Condition: active vs. sham) and one between group factor (Group: able-bodied vs. SCI) to compare changes in gene expression. Statistically significant differences (P<0.05) in gene expression will be corrected for multiple comparisons using the method of Benjamini and Hochberg, with a false discovery rate (FDR)=0.05.

***Hemodynamic stability during RIC.*** A 3-factor mixed model ANOVA will be used to determine differences in HR, BP and SaO _2_ – 2 within group factor (Group; Condition) and one between group factor (Time). The time factor will include: 1) baseline, 2) average of the 5-inflation periods, 3) average of the 5-deflation periods and 4) post-RIC. Significant main or interaction effects will be further explored using Tukey post-hoc analyses. If the data is not normally distributed and the assumptions of the ANOVA are not met, Wilcoxon signed rank tests will be used instead. Post hoc pairwise comparisons will be performed using the Bonferroni adjustment, if there are any main or interaction effects within the two independent variables. The pain scale will be also compared with the same ANOVA model.

### Interim analyses

Not applicable.

### Methods for additional analyses (e.g. subgroup analyses)

Not applicable.

### Methods in analysis to handle protocol non-adherence and any statistical methods to handle missing data

Since this is a crossover study, participants who conduct one but not the second session will probably not be possible to analyze using paired tests, so their data will be excluded.

### Plans to give access to the full protocol, participant level-data and statistical code

No later than three years after data collection, we will deliver a completely deidentified data set to an appropriate data archive for sharing purposes.

## Oversight and monitoring

### Ethics approval and consent to participate

The study protocol has been approved by the institutional review board (IRB) and research and development committee at James J. Peter VA Medical Center. The protocol number is HAR-18-47.

### Composition of the coordinating centre and trial steering committee

Not applicable.

### Composition of the data monitoring committee, its role and reporting structure

This is a short-term single-center pilot study testing a non-invasive intervention with no evidence for serious risk. A data monitoring committee is not needed.

### Adverse event reporting and harms

This clinical trial has multiple levels of data and safety monitoring, beginning with every study personnel involved, extending to the protocol PI, the mentor, a non-affiliated physician safety monitor, the JJPVAMC IRB, the JJPVAMC Information Safety Officer, and all applicable oversight committees on local, state, and national levels.

As detailed in the “Protection of Human Subjects”, this study incorporates many safeguards to exclude participation by subjects at higher risk of adverse events (AEs), and to prevent AEs from occurring in those that are eligible to participate. However, the team knows to always expect the unexpected. Participants will be questioned before, during, and after testing to determine if they have noticed any AE. Any unexpected complications that may occur will lead to immediate termination of that study visit, and discussion with NYH (an experienced neurologist) and the study physician.

All AEs and severe AEs (SAEs) that occur throughout the study, whether study-related or non-related, will be documented and reported to the IRB, as per IRB regulations. AE/SAE report forms will be completed within 48 hours throughout the study for each participant. We will keep a running log of all AE/SAEs (anticipated and unanticipated) throughout the study. Any SAE will be reported to the IRB within 24 hours, and the study will be discontinued until the study physician states that it is safe to resume the study. A formal Data Safety Monitoring Board is not needed for this study. However, depending on the type of SAE or number of SAEs, the investigators and study physician, in consultation with the IRB, could recommend that the study be amended to include a data safety monitoring board, or end earlier than planned. Unanticipated SAEs will warrant suspension of new enrollment until further consultation with the IRB and approval of necessary modifications of the consent and protocol summary to include these risks.

Any significant new information that becomes available during the course of the study that may prevent future related AE/SAEs will be incorporated into the study through appropriate amendments submitted for IRB approval as necessary. Furthermore, if we observe any unanticipated SAEs determined to be related to the study, we will provide oral and written information to update Participants already enrolled in the study, and explicitly remind them of their right to cease participation at any time.

The IRB annually reviews and oversees the risk/benefits within the study. All risks and newly published literature related to this study are reported annually. Ongoing review of the data assures that the study can continue without jeopardizing participant safety.

### Frequency and plans for auditing trial conduct

The study has multiple levels of oversight independent from the investigators and sponsor. In order to comply with federal regulations, research records may be reviewed by the following:

- Representatives of the sponsor JJPVAMC, Center of Excellence for the Medical Consequences of SCI and the SCI Service of this study,
- Authorized representatives of the JJPVAMC (e.g. IRB, Research Compliance Officer), including the Office of Research Oversight
- Federal agencies, such as the Government Accounting Office (GAO), and Food and Drug Administration (FDA)
- The Office for Human Research Protections (OHRP)
- Office of Inspector General (OIG)

The IRB at JJPVAMC will conduct monitoring of source documents and onsite monitoring over the course of the study. They will audit the overall quality and completeness of the data, examine source documents, interview investigators and coordinators, and confirm that the clinical center has complied with the requirements of the protocol. The monitors will verify that all adverse events were documented in the correct format and are consistent with protocol definition.

The monitors will review the source documents as needed, to determine whether the data reported in the Web-based system are complete and accurate. Source documents are defined as medical charts, associated reports and records including initial hospital admission report, etc.

The monitors will confirm that the regulatory binder is complete and that all associated documents are up to date. The regulatory binder should include the protocol and informed consent (all revisions), IRB approvals for all of the above documents, IRB correspondence, case report forms, investigator’s agreements, etc.

If a problem is identified during the auditing (i.e., poor communication with the data coordinating center inadequate or insufficient staff to conduct the study, missing study documents) the monitor will assist the site in resolving the issues.

The focus of the visit/electronic monitoring will be on source document review and confirmation of adverse events. The monitor will verify the following variables for all patients: initials, date of birth, sex, signed informed consent, eligibility criteria, date of randomization, treatment assignment, adverse events, and endpoints

### Plans for communicating important protocol amendments to relevant parties (e.g. trial participants, ethical committees)

All risks and newly published literature related to this study are reported annually to the IRB. Any significant new information that becomes available during the course of the study will be incorporated into the study through appropriate amendments submitted for IRB approval as necessary. Additionally, the institutional review board annually reviews and oversees the risk/benefits within the study. Ongoing review of the data assures that the study can continue without jeopardizing participant safety.

## Dissemination plans

This study is registered at clinicaltrials.gov (NCT03851302). Results will be posted there at study completion.

Dissemination will also occur through the following mechanisms: poster and platform presentations at annual conferences and manuscript publications.

## Discussion

Many approaches to stimulating residual nerve circuits in individuals with SCI have been tested. In this study, we propose a new approach using RIC to transiently impede upper extremity blood flow, to determine if RIC alone, or in combination with task specific exercise training, enhances hand function. Furthermore, we investigate the possible mechanism by examining changes in electrophysiology within the motor cortex and corticospinal tract, and gene expression of inflammatory mediators. Importantly, we will carefully monitor cardiovascular parameters and document pain/discomfort to understand hemodynamic stability and tolerability when applying RIC in persons with cervical SCI. By sharing the details of our NIH-funded research protocol (Grant number: R03HD097709), we hope other interested researchers will seek to investigate similar approaches – depending on overlap with the current study and mutual sharing of participant-level data, this could increase the sample size, power, and generalizability of the analysis and results.

Importantly, given the pilot nature of this study, several limitations are anticipated. First, applying RIC to facilitate motor task learning is a relatively new area with little preliminary data. Therefore, our primary hypothesis that RIC will promote increased corticospinal excitability to hand muscles involved in motor tasks is speculative. Additionally, SCI individuals with neuronal damage to motor and sensory pathways might show diminished responses to RIC on acute post-exercise facilitation compared to non-disabled participants. Heterogeneity in injury level and residual nerve circuits among participants with SCI might result in wide variation in outcomes and we will find some non-responders. Nevertheless, the proposed protocol is designed to provide clear answers. If brief isometric hand exercise plus RIC does not improve corticospinal transmission or pinch strength in participants with cervical SCI, our next step might test RIC in combination with more advanced interventions such as non-invasive paired stimulation, a technique that our research team is actively studying in other protocols (ClinicalTrials.gov Identifier: NCT03414424, NCT02469675 and NCT03806023). Finally, our protocol will test maximal pinch force as the only functional outcome. If the pilot study shows a significant synergistic effect of RIC on the isometric hand exercise task, our future goal will be not only coupling RIC with more prolonged rehabilitation training to promote long-term beneficial effects, but also investigating the benefits in international classification of functioning outcomes.

Additionally, using standard gene expression profiling techniques, if we do not observe changes in gene expression of the TLR signaling pathway, the next step would be to use RNA-sequencing (RNA-Seq) to obtain a broader and unbiased gene expression profile. Initial RNA-seq runs could be limited to 8 million reads/sample, 100bp single end, which would yield a general overview of relative changes in gene expression of the high to moderately expressed genes.

In conclusion, this study is the first to test RIC in people with cervical spinal cord injury and investigate whether RIC alters nerve excitability in the pathway from the brain to the spinal cord to intrinsic hand muscles involved in fine motor tasks. If synergistic effects of RIC with physical training are demonstrated in this study, then effects of RIC coupled with various other rehabilitation interventions can be tested in future studies. In addition, we expect the analysis of neurophysiology and inflammatory mediators before and after RIC to provide some preliminary information regarding the mechanism by which RIC promotes neuroplasticity and improves functional training effects. Thus, results from this study will give us the information to apply RIC coupled with rehabilitation interventions to enhance long-term functional movements in people with cervical spinal cord injury.

## Trial status

The trial was registered on clinicaltrial.gov on February 22, 2019. We started recruitment on November 1st, 2019, and recruitment will be completed on January 31st, 2022.

## Abbreviations

APB: Abductor Pollicis Brevis

FDI: First Dorsal Interosseous

RIC: Remote Ischemic Conditioning

SCI: Spinal Cord Injury

BDNF: Brain-Derived Neurotrophic Factor

AIH: Acute Intermittent Hypoxia

VEGF: Vascular Endothelial Growth Factor

TLR: Toll-like receptor

cDNA: complementary DNA

RT ^2^: reverse transcriptase

PCR: Polymerase Chain Reaction

ANS: autonomic nervous system

HR: Heart Rate

BP: Blood Pressure

SaO _2_: Oxygen Saturation

MEP: Motor Evoked Potential

TMS: Transcranial Magnetic Stimulation

RMT: Resting Motor Threshold

AMT: Active Motor Threshold

MVC: Maximal Voluntary Contraction

ISNCSCI: the International Standards for the Neurological Classification of Spinal Cord Injury

SICI: Short Interval Cortical Inhibition

LICI: Long Interval Cortical Inhibition

ICF: Intra-Cortical Facilitation

CS: Conditioning Stimulus

TS: Test Stimulus

ISI: InterStimulus Intervals

## Data availability

### Underlying data

No data is associated with this article.

### Extended data

Figshare: Informed consent, https://doi.org/10.6084/m9.figshare.14592675.v1 ^51^

### Reporting guidelines

Repository name: SPIRIT checklist for ‘Effects of Remote Ischemic Conditioning on Hand Engagement in individuals with Spinal cord Injury (RICHES): protocol for a pilot crossover study’, https://doi.org/10.6084/m9.figshare.14597403.v2 ^77^

Data are available under the terms of the Creative Commons Zero "No rights reserved" data waiver (CC0 1.0 Public domain dedication).
