# Supplementary material for: Effects of Remote Ischemic Conditioning on Hand Engagement in individuals with Spinal cord Injury (RICHES): protocol for a pilot crossover study
Source: F1000Res. 2022 Mar 7;10:464. Originally published 2021 Jun 10. [Version 2] doi: 10.12688/f1000research.52670.2 (PMC8924555; doi:10.12688/f1000research.52670.2)
Supplement: Peer review history

# Peer review: Reviewer response for version 2

**DOI:** 10.5256/f1000research.119891.r126513

**Published:** 2022-03-15

**Authors:** Andrew Q. Tan

**Affiliations:** 1 Department of Integrative Physiology, University of Colorado, Boulder, CO, USA

The authors have addressed all concerns. I have no further comments.

Is the study design appropriate for the research question?

Yes

Is the rationale for, and objectives of, the study clearly described?

Partly

Are sufficient details of the methods provided to allow replication by others?

Partly

Are the datasets clearly presented in a useable and accessible format?

Not applicable

Reviewer Expertise:

Spinal Cord Injury, Neuromechanics, Locomotion, Sensorimotor Recovery

I confirm that I have read this submission and believe that I have an appropriate level of expertise to confirm that it is of an acceptable scientific standard.

# Peer review: Reviewer response for version 1

**DOI:** 10.5256/f1000research.55978.r88859

**Published:** 2021-08-12

**Authors:** Yo Shih

**Affiliations:** 1 Department of Physical Therapy, University of Nevada Las Vegas, Las Vegas, NV, USA

This study protocol aims to investigate the effect of remote ischemic conditioning on corticospinal excitability, gene expression of inflammatory biomarkers, hemodynamic responses, and functional outcomes. The authors proposed an innovative and well-designed study that will inform future rehabilitation in spinal cord injury.

**Methods**

*Outcomes, Functional outcomes: *The maximal pinch force is the only functional outcome in the study protocol. However, the exercise program is not designed to gain strength as the intensities are submaximal (at 10%, 25%, and 50%). The authors may consider adding accuracy in timing and errors in the level of contraction as part of the functional outcomes so that the “skill” improvement during training could be captured.

*Table 1*: It is helpful to visualize the participant timeline. However, Table 1 could be improved:

1. The “Allocation column” isn’t necessary since it is also at time point 0 and you have a row for Allocation;
2. The items in rows are grouped into three: ENROLMENT, INTERVENTIONS, and ASSESSMENTS. It might be easier to read if the three categories stand out from the rest of the items. A typical timeline figure, similar to Figure 1, may be a better way of demonstration.

I look forward to learn the result of this study.

Is the study design appropriate for the research question?

Yes

Is the rationale for, and objectives of, the study clearly described?

Yes

Are sufficient details of the methods provided to allow replication by others?

Yes

Are the datasets clearly presented in a useable and accessible format?

Yes

Reviewer Expertise:

Neuromechanics, rehabilitation, biomechanics

I confirm that I have read this submission and believe that I have an appropriate level of expertise to confirm that it is of an acceptable scientific standard.

# Response

**Published:** 2021-09-24

**Authors:** Yu-Kuang Wu

**Affiliations:** Bronx Veterans Medical Research Foundation, USA

1. **Outcomes, Functional outcomes: The maximal pinch force is the only functional outcome in the study protocol. However, the exercise program is not designed to gain strength as the intensities are submaximal (at 10%, 25%, and 50%). The authors may consider adding accuracy in timing and errors in the level of contraction as part of the functional outcomes so that the “skill” improvement during training could be captured.**
**Author response: **Thank you very much for this suggestion. We developed a LabVIEW program that provides visual and auditory prompts to participants, so they can follow the prompts to accurately perform the pinch exercise with different submaximal MVC intensity and duration in a pseudo-random submaximal. Please find the video that illustrates how the program works.
https://figshare.com/articles/media/RIC_study_Pinch_exercise_video/16618132
2. **Table 1: It is helpful to visualize the participant timeline. However, Table 1 could be improved:**
**The “Allocation column” isn’t necessary since it is also at time point 0 and you have a row for Allocation;**
**The items in rows are grouped into three: ENROLMENT, INTERVENTIONS, and ASSESSMENTS. It might be easier to read if the three categories stand out from the rest of the items. A typical timeline figure, similar to Figure 1, may be a better way of demonstration.**
**Author response: **The journal modified the format of Table 1. Please find the original format of Table 1 on the Figshare which would be more clear.
https://figshare.com/articles/figure/F1000Research_Table_1/16569120/1
3. **I look forward to learn the result of this study.**
**Author response: **Thank you! We really appreciate your effort to review our manuscript.

# Peer review: Reviewer response for version 1

**DOI:** 10.5256/f1000research.55978.r88145

**Published:** 2021-07-16

**Authors:** Andrew Q. Tan

**Affiliations:** 1 Department of Integrative Physiology, University of Colorado, Boulder, CO, USA

The authors plan an innovative and well-designed proof of concept study on the effects of remote ischemic conditioning (RIC) on hand function after iSCI. This population requires novel therapeutic options to address dosage and retention limitations of current rehabilitation protocols. In a well-written report, the authors describe a protocol to explore the short-term effect of remote ischemic conditioning on pinch force, corticospinal excitability as well as the gene expression inflammatory mediators. The motivation for the study derives from the lack of understanding of both the time course of RIC in the iSCI population and the mechanisms underlying functional gains. Given the lack of available evidence on both the functional and mechanistic changes after iSCI, the testing of this construct is novel with implications for potential clinical translation.

 As the study protocol has been peer reviewed by an external funding body, it perhaps does not require additional peer review. However, please consider addressing the following clarification questions:

- Please state in the funding subsection that the funding body peer reviewed the study protocol.
- Rational could be strengthened by clarifying link between neuroinflammation and study outcomes: TMS amplitude, pinch strength etc. The introduction suggests a putative relationship between RIC induced reductions in inflammation and motor recovery, driven by the rescue of corticospinal excitability. However, this link is not entirely clear to this reviewer. Please clarify the intuition behind this rationale.
- Please resolve seeming inconsistencies in literature cited: Prior literature documents that AIH and high intensity exercise *upregulates *BDNF, while RIC may *attenuate* BDNF expression in the brain. If the expected enhancement of MEP’s is due to *increased* BDNF expression in motor cortex, what is the justification to expect that RIC will enhance CST excitability?  Please clarify equivocal findings.
- The ability of the participants to detect the Sham RIC is well within perceivable range. Consider a blind integrity analyses to account for participant prediction. How will experimenter be blind if they are adjusting the cuff pressure?
- The authors select a homogenous sample of patients with MEPs as part of inclusion criteria for neurophysiological outcomes. This could assume that pinch force gains are primarily due to enhanced excitability.  For motor inclusion criteria, will participants be excluded for those who express tenodesis grip to enhance the pinch force outcome? Furthermore, what methods are employed to avoid floor or ceiling effects in AB SICI/SICF outcomes?
- Task specific training sems to be a critical factor for gaiting RIC induced improvements. However, is it established that RIC *alone* (i.e. no isometric training) does not enhance neuromotor outcomes, especially if the underlying mechanism is currently unknown?
- Are changes in pinch force are sensitive enough to capture immediate yet small effect differences from a single session of RIC given the inherent variability/reliability? This concern is heightened considering the single session/ crossover design where fatigue can influence performance outcomes.  Perhaps there is an analogous clinical assessment that could also be used in conjunction to increase chances of detecting SHAM vs RIC effects. Consider using peak EMG during isometric measurements to validate functional gains.
- Table 1 would benefit from a revision that clarifies which study groups will participate in which protocol and *after *the enrollment process. For example, it is not clear why there are 3 sessions for SCI participants (Sham, RIC, ?). Further, should not gene expression and hemodynamic stability measurements also be marked ‘x’ during allocation column ‘0’ and enrollment column ‘0’ as all enrolled subjects participate in these?

I look forward to the findings of this promising study.

Is the study design appropriate for the research question?

Yes

Is the rationale for, and objectives of, the study clearly described?

Partly

Are sufficient details of the methods provided to allow replication by others?

Partly

Are the datasets clearly presented in a useable and accessible format?

Not applicable

Reviewer Expertise:

Spinal Cord Injury, Neuromechanics, Locomotion, Sensorimotor Recovery

I confirm that I have read this submission and believe that I have an appropriate level of expertise to confirm that it is of an acceptable scientific standard, however I have significant reservations, as outlined above.

# Response

**Published:** 2021-09-24

**Authors:** Yu-Kuang Wu

**Affiliations:** Bronx Veterans Medical Research Foundation, USA

1. **Please state in the funding subsection that the funding body peer reviewed the study protocol.**
**Author response:** We put the funding information in the grant information session: Grant information: National Institute of Health (NIH) [R03HD097709]
2. **Rational could be strengthened by clarifying link between neuroinflammation and study outcomes: TMS amplitude, pinch strength etc. The introduction suggests a putative relationship between RIC induced reductions in inflammation and motor recovery, driven by the rescue of corticospinal excitability. However, this link is not entirely clear to this reviewer. Please clarify the intuition behind this rationale.**
**Author response: **Sorry for the confusion. The rationale is that individuals with SCI sustain chronic inflammation which could possibly attenuate the gene expression of the brain BDNF. RIC has been shown to reduce chronic inflammation which might increase corticospinal excitability. Therefore, we measure the MEP and analyze the gene expression of inflammatory biomarkers before and after RIC.
3. **Please resolve seeming inconsistencies in literature cited: Prior literature documents that AIH and high intensity exercise upregulates BDNF, while RIC may attenuate BDNF expression in the brain. If the expected enhancement of MEP’s is due to increased BDNF expression in motor cortex, what is the justification to expect that RIC will enhance CST excitability? Please clarify equivocal findings.**
**Author response: **Sorry for the confusion. The original sentence “On the other hand, RIC may reduce systemic inflammation, which has been shown to attenuate expression of BDNF in the brain” was trying to state that:
  (a) Lipopolysaccharide-induced systemic inflammation could attenuate gene expression of brain BDNF.
  (b) RIC could reduce systemic inflammation possibly including the protective effects on lipopolysaccharide (LPS)-induced systemic inflammation.
4. **The ability of the participants to detect the Sham RIC is well within perceivable range. Consider a blind integrity analyses to account for participant prediction. How will experimenter be blind if they are adjusting the cuff pressure?**
**Author response: **Due to the nature of the intervention, neither participants nor the research coordinators can be 100% blinded to allocation but are strongly inculcated not to disclose the allocation status of the participant during the experiment. A researcher outside the research team will randomize the order of active and sham RIC for each participant and configure the personal tourniquet system. The researcher who configures the tourniquet pressure would fully cover the pressure indicator.
  Thank you very much for the suggestion to consider a blind integrity analysis. The participants certainly can tell which protocol they have after receiving both active and sham RIC.
5. **The authors select a homogenous sample of patients with MEPs as part of inclusion criteria for neurophysiological outcomes. This could assume that pinch force gains are primarily due to enhanced excitability. For motor inclusion criteria, will participants be excluded for those who express tenodesis grip to enhance the pinch force outcome? Furthermore, what methods are employed to avoid floor or ceiling effects in AB SICI/SICF outcomes?**
**Author response: **As mentioned in the inclusion/exclusion criteria, we conduct the ISNCSCI for each participant with SCI. The participants must show a score of 3 or more (out of 5) on manual muscle testing of finger extension, finger flexion, or finger abduction in the left or right hand. Therefore, we would not enroll the participants using tenodesis grip alone.
  In terms of floor/ceiling effects in AB, we do not have any specific methods applied to the situation. We will definitely discuss the issue when publishing the results if we observe the floor/ceiling effects. We really appreciate the reviewer’s suggestion.
6. **Task specific training seems to be a critical factor for gaiting RIC induced improvements. However, is it established that RIC alone (i.e. no isometric training) does not enhance neuromotor outcomes, especially if the underlying mechanism is currently unknown?**
**Author response: **This is also the question we want to answer in this study, so we conduct a measurement right after active/sham RIC.
7. **Are changes in pinch force are sensitive enough to capture immediate yet small effect differences from a single session of RIC given the inherent variability/reliability? This concern is heightened consi** **dering the single session/ crossover design where fatigue can influence performance outcomes. Perhaps there is an analogous clinical assessment that could also be used in conjunction to increase chances of detecting SHAM vs RIC effects. Consider using peak EMG during isometric measurements to validate functional gains.**
**Author response: **We really appreciate the suggestion of using peak EMG. We do agree that we will not observe any significant pinch force changes after RIC.
  In terms of the fatigue, we design the hand exercise to involve more cortical control (e.g. the participants will need to maintain different certain levels of their peak force (10%, 25%, and 50% MVC) with different duration (2, 4, and 6 secs)) to induce the increase in MEP. We try to see whether active and sham RIC could induce different levels of MEP changes. For the MVC measurement, participants will perform three attempts of maximal pinch force and the best one will be recorded and each time point of the MVC measurement is at least 15 mins apart. We try to prevent the fatigue but the fatigue could still possibly occur.
8. **Table 1 would benefit from a revision that clarifies which study groups will participate in which protocol and after the enrollment process. For example, it is not clear why there are 3 sessions for SCI participants (Sham, RIC, ?). Further, should not gene expression and hemodynamic stability measurements also be marked ‘x’ during allocation column ‘0’ and enrollment column ‘0’ as all enrolled subjects participate in these?**
**Author response: **Sorry for the confusion. The journal modified the format of Table 1. Please find the original format of table 1 on the Figshare: https://figshare.com/articles/figure/F1000Research_Table_1/16569120/1
  As mentioned in the intervention description session. There is a screening session before the allocation of the active or sham RIC session to determine the eligibility of the participants with SCI.
9. **I look forward to the findings of this promising study**
**Author response: **Thank you! We really appreciate your effort to review our manuscript.
